# Genomic Evolution of SARS-CoV-2 Variants of Concern Under In Vitro Neutralising Selection Pressure Following Two Doses of the Pfizer-BioNTech BNT162b2 COVID-19 Vaccine

**DOI:** 10.3390/v17091161

**Published:** 2025-08-25

**Authors:** Kerri Basile, Jessica E. Agius, Winkie Fong, Kenneth McPhie, Danny Ko, Linda Hueston, Connie Lam, David Pham, Sharon C.-A. Chen, Susan Maddocks, Matthew V. N. O’Sullivan, Dominic E. Dwyer, Vitali Sintchenko, Jen Kok, Rebecca J. Rockett

**Affiliations:** 1 Centre for Infectious Diseases and Microbiology Laboratory Services, NSW Health Pathology–Institute of Clinical Pathology and Medical Research, Westmead Hospital, Westmead, Sydney, NSW 2145, Australiadavid.pham@health.nsw.gov.au (D.P.);; 2 Centre for Infectious Diseases and Microbiology–Public Health, Westmead Hospital, Westmead, Sydney, NSW 2145, Australia; 3 The Westmead Institute for Medical Research, Westmead, Sydney, NSW 2145, Australia; 4 Menzies Health Institute Queensland, Griffith University, Southport, QLD 4222, Australia; 5 Sydney Institute for Infectious Diseases, Sydney Medical School, The University of Sydney, Westmead, Sydney, NSW 2145, Australia

**Keywords:** neutralising antibodies, SARS-CoV-2, VOC, B.1.167.2, B.1.351, COVID-19

## Abstract

We aimed to explore SARS-CoV-2 evolution during in vitro neutralisation using next generation sequencing, and to determine whether sera from individuals immunised with two doses of the Pfizer-BioNTech vaccine (BNT162b2) were as effective at neutralising the variant of concern (VOC) Delta (B.1.617.2) compared to the earlier lineages Beta (B.1.351) and wild-type (A.2.2) virus. Using a live-virus SARS-CoV-2 neutralisation assay in Vero E6 cells, we determined neutralising antibody titres (nAbT) against three SARS-CoV-2 strains (wild type, Beta, and Delta) in 14 participants (vaccine-naïve (*n* = 2) and post-second dose of BNT162b2 vaccination (*n* = 12)), median age 45 years [IQR 29–65]; the median time after the second dose was 21 days [IQR 19–28]. The determination of nAbT was based on cytopathic effect (CPE) and in-house quantitative reverse transcriptase real-time quantitative polymerase chain reaction (RT-qPCR) to confirm SARS-CoV-2 replication. A total of 110 representative samples including inoculum, neutralisation breakpoints at 72 h, and negative and positive controls underwent genome sequencing. By integrating live-virus neutralisation assays with deep sequencing, we characterised both functional antibody responses and accompanying viral genetic changes. There was a reduction in nAbT observed against the Delta and Beta VOC compared with wild type, 4.4-fold (*p* ≤ 0.0006) and 2.3-fold (*p* = 0.0140), respectively. Neutralising antibodies were not detected in one vaccinated immunosuppressed participant and the vaccine-naïve participants (*n* = 2). The highest nAbT against the SARS-CoV-2 variants investigated was obtained from a participant who was vaccinated following SARS-CoV-2 infection 12 months prior. Limited consensus level mutations occurred in the various SARS-CoV-2 lineage genomes during in vitro neutralisation; however, consistent minority allele frequency variants (MFV) were detected in the SARS-CoV-2 polypeptide, spike (S), and membrane protein. Findings from countries with high COVID-19 incidence may not be applicable to low-incidence settings such as Australia; as seen in our cohort, nAbT may be significantly higher in vaccine recipients previously infected with SARS-CoV-2. Monitoring viral evolution is critical to evaluate the impact of novel SARS-CoV-2 variants on vaccine effectiveness, as mutational profiles in the sub-consensus genome could indicate increases in transmissibility and virulence or suggest the development of antiviral resistance.

## 1. Introduction

SARS-CoV-2 has continued to circulate globally, with variants of concern (VOC) arising independently in multiple locations worldwide. This ongoing evolution, coupled with a notable increase in the nucleotide substitution rate of emergent variants of concern, suggests that positive selection of advantageous mutations persists within the SARS-CoV-2 genome. These mutations are particularly frequent in the spike (S) glycoprotein, the target for many vaccines and therapeutics [1,2]. Rapid SARS-CoV-2 vaccine development took place, with rollout commencing in 2021, 12 months following the start of the pandemic, and included several different COVID-19 vaccines. In Australia, the Pfizer-BioNTech vaccine (BNT162b2), a nucleoside-modified RNA (mRNA) vaccine targeting the S protein, was primarily given, particularly to healthcare workers from December 2020 [3,4], with similar recommendations worldwide [5]. This timing meant vaccine trials were largely conducted prior to the widespread circulation of VOC, resulting in limited vaccine efficacy data against VOCs in cases of either primary infection or re-infection [6,7]. Subsequent studies have, however, characterised the performance of vaccination against key VOCs, including Delta and Omicron, including the role of booster vaccinations to improve the neutralisation response [8,9,10,11].

The constellation of mutations in the S glycoprotein of different VOCs can reduce the effectiveness of natural and vaccine-induced protection [4,12,13,14,15,16]. The Delta (B.1.617.2) VOC possesses 12 mutations, most notably non-synonymous S mutations L452R, T478K, and P681R, relative to the wild-type SARS-CoV-2, and it lacks markers of convergent evolution such as mutations in S at amino acid positions N501Y or E484K/Q in its angiotensin-converting enzyme 2 (ACE2) receptor-binding domain [17]. T478K has previously been described to decrease susceptibility to monoclonal antibody (mAb) treatment; this mutation has also been reported as acquired during persistent infection in an immunocompromised host [18].

Concerningly, this increased substitution rate coinciding with subsequent waves of infections due to emerging SARS-CoV-2 variants could be due to a variety of selective pressures from natural and vaccine-derived immunity and/or may have emerged through infection in immunosuppressed hosts [18,19]. Hvidt et al. demonstrated cumulative SARS-CoV-2 S mutations were associated with significantly reduced antibody neutralisation capacity [20]. Genomic surveillance of SARS-CoV-2 has been used to monitor for new mutations; however, phenotypic assays are required to predict the impact of these mutations on the efficacy of natural and or vaccine-derived immunity. Whilst live-virus neutralisation remains the gold standard for determining antibody efficacy [21], serum neutralising antibodies (nAb) elicited by vaccination are considered correlates of protection from SARS-CoV-2 disease [8,9,10,11,20,22,23]. There is limited literature, however, on SARS-CoV-2 evolutionary changes associated with evasion of neutralising antibodies, whether infection or vaccination-induced [24].

Reports of rapid evolution of SARS-CoV-2 during propagation in VeroE6 cells have emerged, including the generation of large genomic deletions removing the S1/S2 junction which encodes a putative furin cleavage site [25] and minority allele frequency variants (MFV) [26]. The furin cleavage site primes the Spike protein for cell entry by exposing the S2 fusion peptide to enable virion fusion with the host cell membrane. Transmission of the virus with the cleavage site deletion is attenuated in hamsters and ferrets but outgrows the wild-type virus in VeroE6 cells [27,28]. VeroE6 cells are the predominant cell line used in studies reporting decreases in neutralising antibody titres (nAbT) of SARS-CoV-2 variants in sera from individuals post COVID-19 vaccination. Fold reductions in neutralisation from live-virus micro-neutralisation assays performed in VeroE6 cells occurring due to culture adaptation could therefore be overestimated in these common mutations.

In this study, we assessed vaccine neutralisation titres in vitro using sera collected from Australian healthcare workers who received two doses of BNT162b2, with challenges of live virus infections with the Delta and Beta VOCs compared with the wild-type strain. We combined live-virus neutralisation assays with high-resolution sequencing to monitor both consensus and sub-consensus viral evolution in vitro. In contrast to targeted amplification strategies, our approach enables deeper characterisation of viral changes under neutralising selection pressure or culture adaptation. This knowledge could inform effective public health measures to limit VOC transmission [29,30].

## 2. Materials and Methods

### 2.1. SARS-CoV-2 Culture

Upper respiratory tract specimens were collected in universal transport media (UTM). SARS-CoV-2 RNA was detected by real-time reverse transcriptase polymerase chain reaction (RT-PCR) using either a Cobas^®^ 6800 (Roche Diagnostics GmbH, Mannheim, Germany), a BD MAX™ (Becton Dickinson, Franklin Lakes, NJ, USA), or an in-house assay [31] to inoculate Vero C1008 (Vero 76, clone E6), Vero E6 (ECACC 85020206), or Vero E6 expressing transmembrane serine protease 2 (TMPRSS2) [JCRB1819] cells, as previously outlined [32].

In brief, cells were seeded at 1–3 × 10^4^ cells/cm^2^ whilst in the log phase of replication, with Dulbecco’s minimal essential medium (DMEM) (BE12-604F, Lonza Group AG, Basel, Switzerland) supplemented with 9% foetal bovine serum (FBS) (10099, Gibco™, Thermo Fisher Scientific Inc., Waltham, MA, USA), in Costar^®^ 25 cm^2^ cell culture flasks (430639, Corning Inc., Corning, NY, USA). The medium was changed within 12 h for inoculation media containing 1% FBS and 1% antimicrobials (including amphotericin B deoxycholate (25 μg/mL), penicillin (10,000 U/mL), and streptomycin (10,000 μg/mL)) (17-745E, Lonza Group AG, Basel, Switzerland) to prevent microbial overgrowth and then inoculated with 500 μL of clinical sample into Costar^®^ 25 cm^2^ cell culture flasks. Following inoculation of the clinical sample, all manipulation of SARS-CoV-2 cultures was performed under biosafety level 3 (BSL3) conditions [33].

Cultures were inspected daily for cytopathic effect (CPE); the inoculum and supernatant were sampled at 96 h for SARS-CoV-2 in-house quantitative reverse transcriptase real-time polymerase chain reaction (RT-qPCR) targeting the N-gene, as previously described [34]. A ≥3 cycle decrease in the cycle threshold (Ct) from the inoculum RT-qPCR result (equivalent to a one log increase in viral load) and the presence of CPE were used to determine the propagation of SARS-CoV-2. Viral culture supernatant was harvested 96 h post-infection and a 500 μL aliquot was used to make a SARS-CoV-2 culture bank, where a large volume of passage one stock was made and stored at −80 °C in 500 μL aliquots in 2 mL cryovials (72.694.406, Sarstedt Inc., Nümbrecht, Germany) until required, as detailed in Table S2. SARS-CoV-2 complete genomes were sequenced from the initial clinical specimen, positive culture supernatant, and passage one virus stock to quantify genomic variations that may have developed during propagation (Table S2).

### 2.2. Human Sera Bank

Human sera were sourced from Australian healthcare workers enrolled in the COVID Heroes Serosurvey who were caring for, or handling specimens from, individuals exposed to, or diagnosed with, SARS-CoV-2 infection in Australia [35]. Sera used in this study were collected 19–28 days post-dose two of BNT162b2 vaccination and tested for SARS-CoV-2 antibody titre using an in-house immunofluorescence assay (IFA) against SARS-CoV-2-specific IgA, IgM, and IgG [36] and then stored at −80 °C. Fourteen sera samples were included from an age- and sex-matched cohort of 12 participants (Table S1). These included two vaccine-naïve individuals and 12 individuals who received two doses of BNT162b2 according to the schedule. Median age was 46 years [IQR 29–65] and median time after second dose of vaccine was 21 days [IQR 19–28]. Eleven of the twelve vaccine recipients had no documented history of prior SARS-CoV-2 infection, as confirmed by absence of anti-SARS-CoV-2 nucleoprotein (NP) antibodies, measured by ELISA, on serial sampling since study enrolment prior to COVID-19 vaccination. The remaining vaccine participant had laboratory-confirmed SARS-CoV-2 infection one year prior to vaccination. Serum was heat-inactivated at 56 °C for 30 min prior to microneutralisation.

### 2.3. Determination of 50% Tissue Culture Infective Dose (TCID_50_)

The viral 50% tissue culture infective dose (TCID_50_) was determined for each variant virus. Briefly, a passage one aliquot of virus stock was serially diluted (1 × 10^−2^–1 × 10^−7^) in virus inoculation media. Virus dilutions were used to inoculate Vero C1008 [(Vero 76, clone E6, Vero E6 (ECACC 85020206)] cells at 80% confluence in Costar^®^ 24-well clear tissue culture-treated multiple-well plates (Corning Inc., Corning, NY, USA). Dilutions were seeded in duplicate with two negative (no virus) controls per plate. Plates were sealed with AeraSeal^®^ Film (BS-25, Excel Scientific Inc., Victorville, CA, USA) to minimise evaporation, spillage, and well-to-well cross-contamination. Plates were inspected daily for CPE and 100 μL sampled from each duplicate after inoculation and at 72 h. Infections were terminated at 72 h based on visual inspection for CPE and used in conjunction with RT-qPCR results to determine the TCID_50_ of each isolate.

### 2.4. Micro-Neutralisation Assay

Vero C1008 cells (Vero 76, clone E6, Vero E6 [ECACC 85020206]) were seeded with DMEM (BE12-604F, Lonza Group AG, Basel, Switzerland) from stocks in Costar^®^ 96-well clear tissue culture-treated flat-bottom plates (353072) (Corning Inc., Corning, NY, USA) at 40% confluence. Cells were incubated at 37 °C with 5% C02 for 12 h or until they reached 80% confluence. Virus stocks were diluted to 200 TCID_50_ in inoculation media. Doubling dilutions from 1:10 to 1:320 of vaccine-naïve and post-BNT162b2 vaccination sera were added in equal proportions with virus in a 96 well plate and incubated for 60 min at 37 °C 5% C02 to enable virus neutralisation. The medium was then removed from the cell monolayer and 100 μL of fresh medium was added. Each dilution of sera was performed in duplicate per virus variant, 12 wells of uninfected cells were used on each plate as a negative control. Plates were sealed with AeraSeal^®^ Film to minimise evaporation, spillage, and well-to-well cross-contamination. After 60 min of viral neutralisation, a residual 110 μL was sampled from the 12 naïve-patient wells per virus for extraction and RT-qPCR. The plates were inspected daily for CPE with a final read recorded independently by two scientists at 72 h. SARS-CoV-2 in-house RT-qPCR was used to quantify the viral load post-neutralisation, with 110 μL of each dilution removed at 72 h to determine viral load. The 110 μL of each dilution was added to 110 μL of External Lysis buffer (06374913001, Roche Diagnostics GmbH, Mannheim, Germany) at a 1:1 ratio in a 96-well deep-well extraction plate (Roche Diagnostics GmbH), covered with MagNA Pure Sealing Foil (06241603001, Roche Diagnostics GmbH, Mannheim, Germany), and left to rest in a biosafety class two cabinet for 10 min, a time-period shown to inactivate SARS-CoV-2 by in-house verification of a published protocol [37]. The RNA was then extracted with a Viral NA Small volume kit (06 543 588 001, Roche Diagnostics GmbH, Mannheim, Germany) on the MagNA Pure 96 system (Roche Diagnostics GmbH, Manheim, Germany).

### 2.5. SARS-CoV-2 Genome Sequencing Following Respiratory Viral Oligo Panel Enrichment

A total of 110 samples underwent SARS-CoV-2 whole genome sequencing. These included RNA extracts collected 1 h post-neutralisation (replicates of naïve 1:10 sera neutralisation) representing the baseline viral inoculum and neutralisation breakpoints as defined by CPE for each sera tested 72 h post-neutralisation. Both biological replicates of each breakpoint were included, as were replicates of the naïve neutralisation at the highest sera dilution (1:10 and 1:20). Five specimens collected 72 h after neutralisation from uninfected wells on each plate were used as negative controls. A synthetic RNA SARS-CoV-2 construct (TWIST Biosciences, San Francisco, CA, USA) containing the reference SARS-CoV-2 sequence (National Center for Biotechnology Information (NCBI) GenBank accession MN908947.3) was diluted in negative control RNA (1:10) and was included in triplicate to control for library preparation and sequencing artefacts.

**Figure 1:**
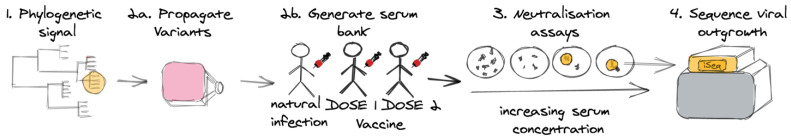
Outline of virus neutralisation assays of emerging SARS-CoV-2 variants of concern.

Viral enrichment was performed using the Illumina RNA Prep with the Respiratory Viral Oligo Panel version 2 (RVOP) (Illumina Inc., San Diego, CA, USA) (Figure 1). RNA extracts from the microneutralisation and TCID_50_ experiments were used as input into the RNA Prep with Enrichment kit (Illumina Inc., San Diego, CA, USA). RNA denaturation, first- and second-strand cDNA synthesis, cDNA tagmentation, library MFV construction, clean up, and normalisation were performed according to manufacturer’s instructions. Individual libraries were then combined in 3-plex reactions for probe hybridisation. The RVOP was used for probe hybridisation with the final hybridisation step held at 58 °C overnight. Hybridised probes were then captured and washed according to manufacturer’s instructions and amplified as follows: initial denaturation: 98 °C for 30 s, 14 cycles of: 98 °C for 10 s, 60 °C for 30 s, 72 °C for 30 s, and a final 72 °C for 5 min. Library quantities and fragment size were determined using Qubit™ 1x dsDNA HS Assay (Invitrogen–ThermoFisher Scientific Inc., Waltham, MA, USA) and Agilent HS D1000 Screentapes (Agilent Technologies Inc., Santa Clara, CA, USA). Resulting libraries were pooled with the aim of generating 1 × 10^6^ raw reads per specimen and sequenced producing paired 74 base-pair reads on the Illumina MiniSeq or iSeq instruments (Illumina Inc., San Diego, CA, USA) (Figure 1).

### 2.6. Bioinformatic Analysis

Raw sequence data were processed using an in-house quality control procedure prior to further analysis. De-multiplexed reads were quality trimmed using Trimmomatic v0.36 (sliding window of 4, minimum read quality score of 20, leading/trailing quality of 5 and minimum length of 36 after trimming) [38]. Briefly, reads were mapped to the reference SARS-CoV-2 genome (NCBI GenBank accession MN908947.3) using Burrows–Wheeler Aligner (BWA)-mem version 0.7.17 [39], with unmapped reads discarded. Average genome coverage was estimated by determining the number of missing bases (N’s) in each genome. Variants were called using VarScan v 2.3.9 [40] (minimum read depth > 10×, quality > 20 min. frequency threshold of 0.1). Single nucleotide polymorphisms (SNP)s were defined based on an alternative frequency ≥0.75 whereas MFV were defined by an alternative frequency between 0.1 and 0.75. Variants falling in the 5′ and 3′ UTR regions were excluded due to poor sequencing quality of these regions. Polymorphic sites that have previously been highlighted as problematic were monitored and annotated in the results [41]. To ensure the accuracy of variant calls, only high-quality genomes with greater than 99% genome coverage and a median depth of 200× were included. The MFV calls were excluded in the base pair either side of the 5′ or 3′-end of indels due to miss-mapping. SARS-CoV-2 lineages were inferred using Phylogenetic Assignment of Named Global Outbreak LINeages v1.36.8 (PANGOLIN) [42]. Graphs were generated using RStudio (version 3.6.1).

### 2.7. Statistical Analysis

Mean nAbT values were evaluated and statistical significance assessed using the *t*-test with a 2-tailed hypothesis. Results were considered statistically significant at *p* < 0.05.

## 3. Results

### 3.1. Levels of Neutralising Antibody Against Different SARS-CoV-2 Lineages

**Figure 2:**
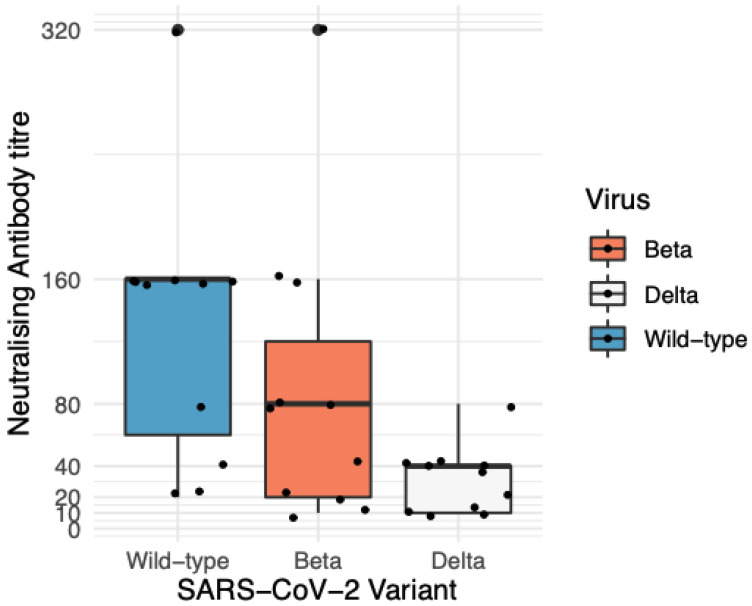
Neutralising antibody titres against tested SARS-CoV-2 lineages. Differences in neutralising antibody titres (nAbT) against the wild-type, Beta, and Delta SARS-CoV-2 lineages, median of 21 days [IQR 19–28] after receiving the second dose of Pfizer-BioNTech (BNT162b2) vaccine, measured by visual inspection of cytopathic effect (CPE) with confirmation of viral replication by SARS-CoV-2 by in-house RT-qPCR. Results are reported in the box−whiskers plots as medians and upper and lower quartiles. There was a significant fold reduction in nAbT observed between both the Delta [(M = 4.4, SD = 2), t(11) = −4.9, *p* = 0.00059], and Beta [(M = 2.3, SD = 2) t(11) = 3, *p* = 0.01397] VOCs compared with wild-type. There was also a significant fold reduction in nAbT between Beta [(M = 2.6, SD = 1.4), t(11) = −2.5, *p* = 0.02897 and Delta. Key: Delta—Delta B.1.617.2] lineage; Beta—Beta B.1.351 lineage; Wild-type—wild-type A.2.2 lineage; RT-qPCR—reverse transcriptase real-time quantitative polymerase chain reaction.

Genomic sequencing results indicated that the samples sequenced were wild-type strain (lineage A.2.2), Beta (lineage B.1.351), or Delta (lineage B.1.617.2) VOC. Following-vaccination with two doses of BNT162b2, 11 of 12 recipients demonstrated a functional neutralisation response to wild-type virus (Table S1 and Figure 2). The serum from an immunosuppressed participant that failed to mount a serological response to wild-type virus post-vaccination was excluded from further analysis to calculate fold reductions in nAbT (Table S1). No detectable antibodies or functional neutralisation responses were seen in sera collected prior to vaccination (*n* = 2) (Table S1).

The median nAbT in BNT162b2 vaccine recipients who responded (*n* = 11) when challenged with wild-type virus was 160 (range < 10–320), compared with 80 (range < 10–320) and 40 (range < 10–80) for Beta and Delta, respectively (Figure 2). There was a significant fold reduction in nAbT observed between both the Delta [(M = 4.4, SD = 2), t(11) = −4.9, *p* = 0.00059), and Beta ((M = 2.3, SD = 2), t(11) = 3, *p* = 0.01397] compared with the wild type (Figure 2). There was also a significant fold reduction in nAbT between Beta [(M = 2.6, SD = 1.4), t(11) = −2.5, *p* = 0.02897] and Delta (Figure 2).

Participants aged ≤45 years had significantly higher fold reductions in nAbT (M = 5.6, SD = 2.2) compared with those aged over 46 (M = 3.3, SD = 1) between the wild-type and Delta strains, t(5) = 4.9, *p* = 0.00788. The ≤45 years cohort also had significantly higher fold reductions in nAbT (M = 4, SD = 0) compared with >46 yrs (M = 1.4, SD = 0.7) between Beta and Delta strains, t(5) = 3.2, *p* = 0.03397. No significant sex specific effects were identified. The highest nAbT for all SARS-CoV-2 variants investigated was obtained from a participant who was infected with SARS-CoV-2 one year prior to vaccination (Table S1).

### 3.2. Quantification of Inoculation Dose for Different Lineages of SARS-CoV-2

**Figure 3:**
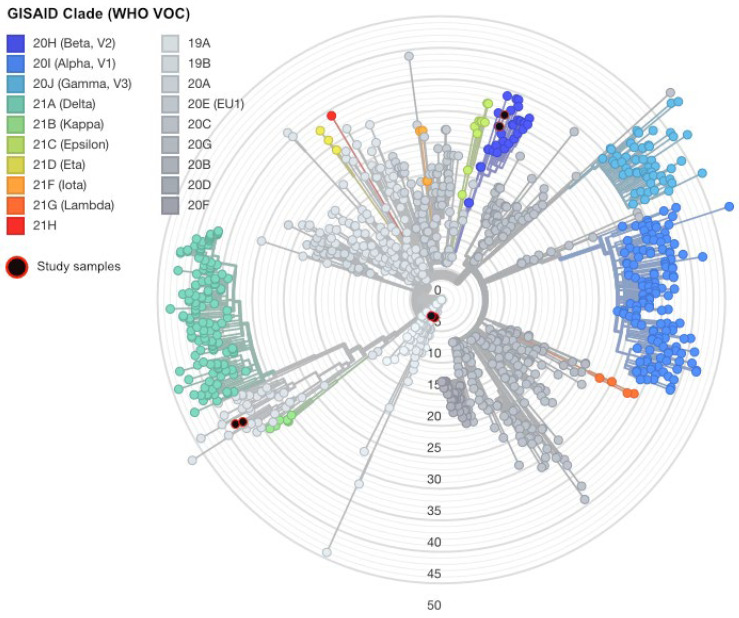
Global SARS-CoV-2 diversity demonstrating representativeness of inoculating viruses used in this study. The genome sequence of the lineages neutralised by sera post-2 doses of vaccination with BNT162b2 were included in the subsampled global phylogeny of SARS-CoV-2. Representative sequences were selected by Nextstrain and used to generate a global phylogeny. The original clinical specimen and inoculating virus sequence is highlighted in black with red outline. Key: VOC—variant of concern; WHO—World Health Organization.

The mutational profile of the inoculum (Tables S2 and S3) for each lineage was consistent with the mutational profile defining the PANGO lineages assigned to the original clinical specimen (Figure 3). The SARS-CoV-2 genomes of the inoculated viruses, the original clinical specimens, and viruses isolated in cell culture were compiled with representation of the global SARS-CoV-2 diversity (*n* = 1000) curated by Nextstrain (https://nextstrain.org/) (Figure 3). The RT-PCR Ct value of the inoculum of the wild-type and Beta VOC was 28, whereas the Ct of the Delta VOC was 24. This difference in Ct values could be explained by the increased sensitivity of PCR assays; however, PCR is unable to differentiate between infectious SARS-CoV-2 virions and non-viable virus.

### 3.3. SARS-CoV-2 Polymorphism in Culture

A total of 110 samples underwent SARS-CoV-2 sequencing including 102 extracts from the microneutralisation experiment, five negative controls, and the three SARS-CoV-2 synthetic constructs used as positive controls (TWIST Biosciences, encoding NCBI GenBank Accession MN908947.3). All but one genome was recovered with high read depth [average depth 3300.5× (range 174–11,844)] and the average genome coverage was 99.97% (range 99.73–100%). The five negative control samples contained <10 SARS-CoV-2 specific reads.

A total of 3039 polymorphisms were detected during neutralisation (2715 majority allele variants and 324 minority allele frequency variants (MFVs)), and the most frequent base change was C>U (Figure S2). We focused our investigation on genomic variants that were not in the viral inoculum and developed 72 h post-neutralisation (majority allele variants = 21 and MFV = 176). Base change dynamics were similar between majority variant polymorphisms, de novo consensus level changes, and de novo MFV, apart from G>C changes noted at high frequency in the de novo MFV (Figure S2). Non-synonymous mutations were detected at greater frequency than synonymous, indels, or nonsense mutations. A higher ratio of non-synonymous to synonymous (Ka/Ks) mutations was detected when comparing de novo MFV (Ka/Ks 3.35) to majority allele variants (Ka/Ks 1.38) (Figure S2).

Aliquots of the virus–serum inoculum were collected 1 h post-neutralisation in biological replicates, for each SARS-CoV-2 variant investigated. The consensus and MFV mutations were tabulated and used as a baseline for our analysis (Table S3).

No additional mutations or MFVs were noted in the viral inoculum within the furin cleavage site, previously reported after long-term passage in Vero E6 cells (Table S3) [25,43,44,45,46,47,48].

### 3.4. Persistence and Conversion of Minority Frequency Allele Variants 72 h Post-Neutralisation

**Figure 4:**
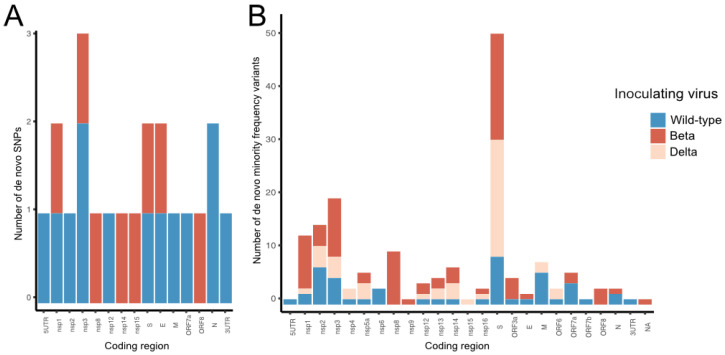
Frequency of consensus and minority allele frequency variants within the SARS-CoV-2 genome. Count of de novo consensus (**A**) and minority frequency allele variants (MFVs) (**B**) within SARS-CoV-2 genes 72 h post-neutralisation. Consensus and MFVs detected in the inoculating virus of each lineage have been excluded. Although few consensus level mutations were detected 72 h post-neutralisation, a high number of MFV were detected within the spike coding region. Key: structural proteins [S—spike; E—envelope; M—membrane and N—nucleocapsid]; nsp—non-structural protein; ORF—open reading frame; 5UTR—5′ untranslated region, 3UTR—3′ untranslated region, Delta—Delta B.1.617.2 lineage; Beta—Beta B.1.351 lineage; Wild-type—Wild-type A.2.2 lineage.

Minimal consensus level mutations were noted 72 h post-neutralisation compared with the viral inoculum (Figure 4 and Figure S3); however, sub-consensus MFV persisted in Beta and Delta infections (Figure S3). Persistence of MFVs at position C13667T (nsp12, orf1ab p.4468T>I) detected at a read frequency of 0.06–0.22 was noted in 32/33 biological replicates, including the inoculating virus. A total of six MFVs were detected in the Beta inoculum, four of which persisted and generally increased in frequency 72 h post-neutralisation. The MFV at position C11249T (nps6, orf1ab p.3662R>C) was detected 1 h post-neutralisation at an average frequency of 0.53, the mutation persisted in 26/32 replicate infections at 72 h and developed into a consensus base change in 13/26 replicates. The MFV at position C11750T (nsp6, orf1ab p.3829L>F) was detected 1 h post-neutralisation at a median frequency of 0.25 and persisted in 20/32 infections at 72 h, developing into consensus mutations in six infections. Synonymous mutations at C27911T (orf8 p.6F) were detected at an average frequency of 0.065 1 h post-neutralisation, and persisted in 9/32 infections, converting to a consensus mutation in a single infection. A second synonymous MFV at position C29077T (N:p.268Y) was detected at a frequency of 0.66 1 h post-neutralisation and was detected in 27/32 infections at 72 h, converting to a SNP in 12/27 infections. However, the two MFVs detected 1 h post-neutralisation in the wild-type variant (G18670T, nsp14, orf1ab p.6136D>Y, G22316A S: p.252G>S) were detected at a low read frequency (*p* < 0.01) and did not persist in any infections at 72 h.

### 3.5. De Novo Majority Frequency Variants

De novo mutations that were not detected 1 h post-neutralisation were also investigated (*n* = 21, Figure 4 and Figure S2). When the wild-type virus (A.2.2) was neutralised, a maximum of three consensus-level mutations developed in any infection, compared with the sequence of the inoculum (median 0, range 0–3) (Figure 3 and Figure 4, Table S3). Of 11 consensus-level mutations within the coding region, 6 were synonymous and 5 were non-synonymous (Figure S2). Only eight de novo consensus level mutations were detected 72 h post-neutralisation after inoculating with the Beta variant. A maximum of two consensus level mutations developed in any infection compared to the inoculum sequence (median 0, range 0–1). None of the genomic positions detected were replicated over the 32 infections. No consensus level mutations developed 72 h post infection when Delta was neutralised in the 34 infections.

### 3.6. Development of De Novo Minority Allele Frequency Variants

**Figure 5:**
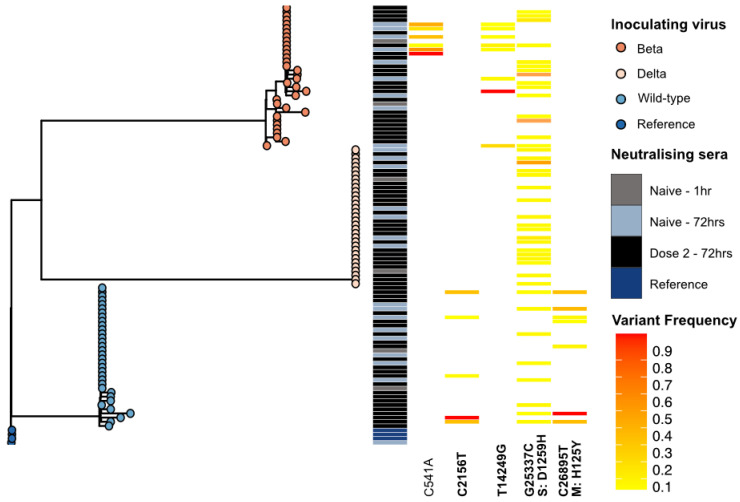
Phylogeny of SARS-CoV-2 diversity during neutralisation highlighting minority variants reproducibly detected at the same genomic location. A maximum likelihood phylogeny of the 109 SARS-CoV-2 genomes generated in this study. Tree node colours indicate the lineage of the inoculating virus and the heatmap highlights the time of sampling and the vaccine dose of the sera used. De novo minority allele frequency variants (MFVs) that were consistently detected (≥5 biological replicates) at specific genomic locations are shown in the heatmap. The read frequency of these sub-consensus variants is depicted by the colour scale, where a frequency of 0.1 is shown in yellow and a frequency of 0.9 is shown in red. The MFVs detected in the inoculating viruses are not included. Reference SARS-CoV-2 genome: NCBI GenBank accession MN908947.3. Key: Delta—Delta (B.1.617.2) lineage; Beta—Beta (B.1.351) lineage; Wild-type—wild-type A.2.2 lineage; 72 h—samples collected at 72 post-neutralisation, Clinical sample—from the original patient’s sample used to generate the culture isolate.

MFVs were generally homogeneously detected across the SARS-CoV-2 genome; however, a concentration of MFVs in the S protein was noted (Figure 4). Novel MFVs (*n* = 63) were detected at 32 unique genomic positions 72 h post-neutralisation of the wild-type virus (Figure 4). Three of these genomic locations (C2156T nsp2 orf1ab p.631L>F, G25337C S:p.1258D>H, C26895T M:p.125H>Y) were reproducibly detected in >5 infections (Figure 5). The MFV converted to a consensus change at position C2156T in a single infection.

Novel MFVs (*n* = 86) were detected in 50 unique positions 72 h post-neutralisation of the Beta VOC. Three genomic positions [(C541A nsp1 orf1ab p.92L), (T14249G nsp8, orf1ab p.4662L>W), (G25337C S:p.1258D>H)] were reproducibly detected in >5 biological replicates. The MFV converted to a consensus change at position T14249G in a single infection.

When the Delta VOC was sequenced 72 h post-neutralisation, novel MFVs (*n* = 48) were detected at 28 unique genomic positions. A single MFV at position G25337C was repeatedly detected in 18/32 infections.

A high frequency of MFV in the S protein coding region were detected during genome-wide analysis. Of the 51 MFV detected in the S protein 72 h post-neutralisation with any SARS-CoV-2 virus, 41/51 were detected at position 25337, which encodes a non-synonymous mutation D1259H in the C-terminal domain of the S protein. This mutation was not detected in the genomes generated one hour post-neutralisation. Only three MFVs were detected within the S1/S2 cleavage site at nucleotide positions 23606 (S p.682R>W), 23616 (S p.691S>A), and 23633 (S p.697M>T) at read frequencies of 0.05, 0.09, and 0.08, respectively.

The only MFV that persisted across all lineages was at position G25337C. This variant was detected in 41/102 infections but was not detected in the viral inoculum. This MFV persisted at a low frequency (median 0.08 min 0.05 max 0.15).

Indels detected in the minority of reads were also uncovered in 9/102 infections, generating six deletions and four insertions. A 10bp deletion was detected at nucleotide position 685 (AAAGTCATTT685A, orf1ab p.141-144KSFD>X) 72 h after the Beta VOC was neutralised in two infections, at a read frequency of 0.06 and 0.17. Two additional deletions were detected in nsp1 at positions 514 and 515 [(TGTTATG515T orf1ab p.84-85VM>X) and (GTTA515G orf1ab p.84-85VM>-)]. Single base insertions and deletions were identified in single infections [(5/102 G1772GT orf1ab p.503V>F), (TG16911T orf1ab p.5550V>X), (T25878TC ORF3a p.162-163->X), (GA27396G ORF7a p.2K>X) (G27906GT ORF8 p.5V>VX)].

## 4. Discussion

In this study, we confirm a reduction in nAbT after BNT162b2 vaccination when challenged with the Beta VOC [49,50,51], and demonstrate a significant 4.4-fold reduction in nAbT against the Delta VOC. Herein, we highlight the higher fold reduction in nAbT against the Delta compared to the Beta VOC. We demonstrate that serum from a vaccine recipient who was previously infected with SARS-CoV-2 mounted the highest nAbT against all virus variants [52], and that immunocompromised individuals may fail to mount neutralising antibody responses despite completing a two-dose BNT162b2 vaccination schedule [53]. This finding, while limited by sample size, is consistent with the reported literature.

To ensure the validity of our findings and to control for genomic adaptations in the furin cleavage site, commonly reported when SARS-CoV-2 is cultured in Vero E6 cells, we undertook genomic analysis of the viral inoculum and outgrowth compared with the original clinical isolate.

At 72 h post-neutralisation, limited de novo consensus mutations were noted in comparison to the infecting VOC. However, several de novo MFVs were detected in the inoculum and persisted in biological replicates post-neutralisation, demonstrating the utility of deep sequencing and hybridisation probe capture [54] to accurately monitor MFVs. The higher resolution provided by sequencing enabled accurate monitoring of MFVs, but the method is potentially confounded by homoplasy in the SARS-CoV-2 genome, transmission bottlenecks, and the transient nature of many MFVs during the course of infection [54,55,56].

With one exception, these genomic locations were not conserved between infecting viral lineages, and the genomic positions were not in identified homoplastic sites. One MFV in the C-terminal of S was detected in 41/96 infections 72 h post-neutralisation. This MFV resulted in a D1258H mutation, which had been previously reported in only 50 SARS-CoV-2 consensus sequences available on the Global Initiative on Sharing All Influenza Data (GISAID) EpiCoV database as of 12 November 2021 [57]. Rocheleau et al. reported the detection of this MFV in both clinical and cultured SARS-CoV-2 genomes and provided evidence that missense mutations that truncate the C-terminal domain of the S protein enable more efficient viral exocytosis by promoting direct cell–cell fusion [58]. The reproducible detection of this MFV requires further investigation to determine whether it provides a selective advantage, or whether this genomic site is homoplastic.

The significance of SARS-CoV-2 diversity driven by de novo mutations within hosts has been recognised as important, particularly as evidence of positive selection of mutations that can evade immunity and therapeutics and have been demonstrated in immunocompromised patients and in in vitro systems [19,54,59]. Despite this, booster doses of SARS-CoV-2 vaccination can significantly improve neutralising antibody titres, even against some VOCs [9,10,11,20]. Furthermore, the development of variant-specific vaccinations offers increased effectiveness over boosting with ancestral virus-derived vaccines [29,60,61,62]. The epidemiology of SARS-CoV-2 remains constantly in flux, and varying levels of seasonal natural infection, vaccine coverage, and variant evolution, all contribute to the complexity of public health policy and optimal vaccination strategies, including boosting and the use of ancestral-based and variant-specific vaccines [24].

It is important to note that our controlled in vitro system may have limited generalisability. The MFVs observed may represent artefacts of culture adaptation in VeroE6 cells or technical noise rather than true immune escape pathways. Furthermore, the simplified conditions of in vitro assays do not replicate the complex selective pressures present in natural infection, such as prior immune history, transmission bottlenecks, and host diversity. These in vitro phenotyping systems can provide valuable insights into viral dynamics; however, mutational profiles identified in the sub-consensus genome should be interpreted with caution, as they may not directly translate to vaccine escape or population-level viral evolution without supporting clinical and epidemiological data. Such systems will require adequate controls for mutations driven by culture adaptation that may not correlate with virus evolution in vivo. Nevertheless, our approach provides an important model for understanding potential population dynamics of SARS-CoV-2 infections, as mutational profiles in the sub-consensus genome may illuminate variants with increased transmissibility, virulence, and/or antiviral and mAb resistance.

Despite the relatively low number of participants studied herein, the cohort included age- and sex-matched participants immunised with two doses of BNT162b2 exactly three weeks apart and a median time to serum collection of 21 days post-dose two [IQR 21–29] when peak antibody production post-vaccination would be expected. The lack of detectable antibodies in pre-vaccination sera reflects the relatively low incidence of COVID-19 infection and initial slow vaccine uptake in the Australian population [63].

## 5. Conclusions and Future Directions

The rapid global adoption of genomic sequencing combined with traditional epidemiology to address the COVID-19 pandemic has enabled real-time surveillance of SARS-CoV-2 evolution. The ability to determine the frequency of cases with specific mutational profiles has provided strong indicators of SARS-CoV-2 variants with selective advantage [64,65,66]. Supporting evidence from in vitro studies, and SARS-CoV-2 coding positions demonstrating convergent evolution, has clearly highlighted S protein mutations that have increased infectivity or transmissibility [28,48].

Our findings demonstrated a significant reduction in nAb against the Delta compared to Beta and wild-type variants. Modelling has predicted that a five-fold decrease in nAbT would likely reduce the effectiveness of current vaccines from 95% to 77% for high efficacy vaccines and 70% to 32% for lower efficacy vaccines [67]. Deep sequencing and hybridisation probe capture constitute an approach less hampered by amplification biases, enabling high-resolution monitoring of SARS-CoV-2 evolution including mutational profiles in the sub-consensus genome. This increased resolution may provide useful insights into vaccine effectiveness when interpreted alongside epidemiological and clinical data. However, in vitro findings should not be over-generalised, and confirmation in population-level and clinical studies is required before attributing mutational profiles to transmissibility, virulence, or resistance.

Further studies assessing convalescent serum samples and other markers of immunity to determine correlates and duration of protection against emerging variants of interest and VOCs, as well as assessing nAbT responses in serially collected serum samples are warranted. Similar approaches could also be used to test sera from individuals receiving vaccines other than BNT162b2 (e.g., other mRNA, viral vector, protein or inactivated SARS-CoV-2 vaccines) [68]. Comparisons between primary and booster vaccinations should also be investigated to determine the optimal vaccination strategy at both individual (including those that are immunocompromised) and population levels.

## Data Availability

The data presented in this study are available on request from the corresponding author due to privacy reasons.

## Supplementary Materials

The following supporting information can be downloaded at: https://www.mdpi.com/article/10.3390/v17091161/s1, Figure S1: Change in SARS-CoV-2 viral load three days post-neutralisation in vaccine responders and non-responders; Figure S2: Mutational analysis of fixed mutations and minority allele frequency variants during live virus in vitro neutralisation; Figure S3: Evolution of minority frequency variants in inoculating viruses 72 h post-neutralisation; Table S1: Study Participant Demographic and Inhouse SARS-CoV-2 Immunofluorescence (IFA) and Neutralising Antibody Titres (nAbT); Table S2: Summary of Viral Stock used; Table S3: Mutational profile of inoculum.
