# Supplementary material for: Genomic Evolution of SARS-CoV-2 Variants of Concern Under In Vitro Neutralising Selection Pressure Following Two Doses of the Pfizer-BioNTech BNT162b2 COVID-19 Vaccine
Source: Viruses. 2025 Aug 25;17(9):1161. doi: 10.3390/v17091161 (PMC12474427; doi:10.3390/v17091161)
Supplement: Supplementary file 1 [file viruses-17-01161-s001.zip › Supplementary Material_SM_DPaccepted_RR.pdf]

Supplementary Table S1: Study Participant Demographic and Inhouse SARS-CoV-2 Immunofluorescence (IFA) and Neutralising Antibody Titres (nAbT)

	Sex	Age (yrs)	Post 2nd dose BNT162b2 (days)	SARS-CoV-2 IFA			Lineage			Other
				IgG	IgA	IgM	Wild-type	Beta	Delta	
**1**	F	36	21	1280	<10	<10	160	80	20	
**3**	M	49	28	320	<10	<10	20	<10	<10	
**4**	M	29	22	640	10	<10	80	40	<10	
**5**	M	65	22	640	<10	<10	160	20	40	
**6**	F	35	21	1280	10	<10	320	320	80	Previously infected with SARS CoV-2
**7**	F	44	19	<10	<10	<10	<10	<10	<10	Immunosuppressed
**8**	F	45	21	640	<10	<10	40	20	<10	
**9**	F	59	21	160	<10	<10	20	<10	<10	
**10**	F	36	21	640	<10	10	160	160	40	
**11**	F	57	21	1280	<10	<10	160	80	40	
**12**	M	43	28	1280	<10	10	160	160	40	
**13**	M	47	22	1280	<10	<10	160	80	80	
**4**	M	29	naïve	<10	<10	<10	<10	<10	<10	Naïve pre-vaccination sera
**11**	F	57	naïve	<10	<10	<10	<10	<10	<10	Naïve pre-vaccination sera

Key: Beta – Beta (B.1.351) lineage; BNT162b2 – Pfizer-BioNTech (BNT162b2) vaccination; Delta – Delta (B.1.617.2) lineage; F – female; IFA –Immunofluorescence Assay; IgA – immunoglobulin A; IgG – immunoglobulin G; IgM – immunoglobulin M; M – male; Wild-type – Wildtype (A.2.2) lineage.

Supplementary Table S2: Summary of Viral Stock used.

Lineage	WHO Variant of Concern	GISAID ID of original clinical isolate	Cells originally isolated in	Passage number
A.2.2	-	hCoV-19/Australia/NSW183/2020	Vero C1008 (Vero 76, clone E6, Vero E6 [ECACC 85020206]	1
B.1.351	Beta		TMPRSS2 expressing VeroE6 [JCRB1819]	1
B.1.617.2	Delta	hCoV-19/Australia/NSW1601/2021	TMPRSS2 expressing VeroE6 [JCRB1819]	1

Key:
GISAID – Global initiative on sharing all influenza data;
Lineage – PANGO Lineage_ version 3;
TMPRSS2 – transmembrane serine protease 2.

Supplementary Table S3: Mutational profile of inoculum

**Inoculating virus**	**Consensus mutations (Protein changes)**	**Low frequency variants (read frequency, protein changes)**
**Wild type (Lineage A.2.2)**	C4540T, C8782T, T9477A (nsp4, F308Y), C14805T, G25979T (ORF3a, G196V), T28144C (ORF8, L84S), C28311T (N, P13F), C28657T, C28863T (S197L)	G18670T (6.08%*, nsp14, D211Y), G22316A (5.37%*, S, G252S)
**Beta (Lineage B.1.351)**	G174T, C241T, C1059T (nsp2, T85I), C3037T, T4213C, G5230T(nsp3, K837N), A10323G (nsp5, K90R), C11812A, GTCTGGTTTT11287G, C14408T (nsp12, P323L), C15925T (nsp12, L829F), T17970C, A20662G (nsp16, S2G), G21641T (S, A27S), A21801C (S, D80A), A22206G (S, D215G), ACTTTACTTG22280A, G22813T (S, K417N), G23012A (S, E848K), A23063T (S, N501Y), A23403G (S, D614G), C23664T (S, A701V), A23764T, G25563T (ORF3a, Q57H), C25904T(ORF3a, S171L), C26456T (E, P71L), C28253T, T28642C, G28871T (N, G200C), C28887T (N, T205I)	C11249T (49%, nsp6, R97C), C11750T (25%, nsp6, L206F), C12114T (12%*, nsp8, S8F), T14637A (11%*), A27452G (5%*, ORF7a, Y20C), C27911T (7%), C29077T (67%)
**Delta (Lineage B.1.617.2)**	G210T, C241T, C1191T (nsp2, P129L), C1267T, C3037T, G4201T (nsp3, M494L), C5184T(nsp3, P822L), C6539T (nsp3, H1274Y), C9891T (nsp4, A446V), T11418C (nsp6, V149A), T12946C, C14408T (nsp12, P323L), T15225C, G15451A (nsp12, G671S), C16466T (nsp13, P77L), A20262G, C20320T (nsp15, H234Y), C21618G (S, T19R), G21793T (S, K77B), G21987A (S, G142D), GAGTTCA22028G, C22591T, T22917G (S, L452R), C22995A (S, T478K), A23403G (S, D614G), C23604G (S, P681R), G24410A (S, D950N), C24745T, C25469T (ORF3a, S26L), C26353T (E, L37F), T26767C (M, I82T), T27638C (ORF7a, V82A), C27739T (ORF7a, L116F), C27752T (ORF7a, T120I), AGATTTC28247A, TA28270T, A28461G (N, D63G), G28881T (N, R203M), G29402T (N, D377Y), G29427A (N, R385K), G29742T	C13667T (14.18%, nsp12, T76I)

Supplementary Figure S1: Change in SARS-CoV-2 Viral load three days post-neutralisation in vaccine responders and non-responders

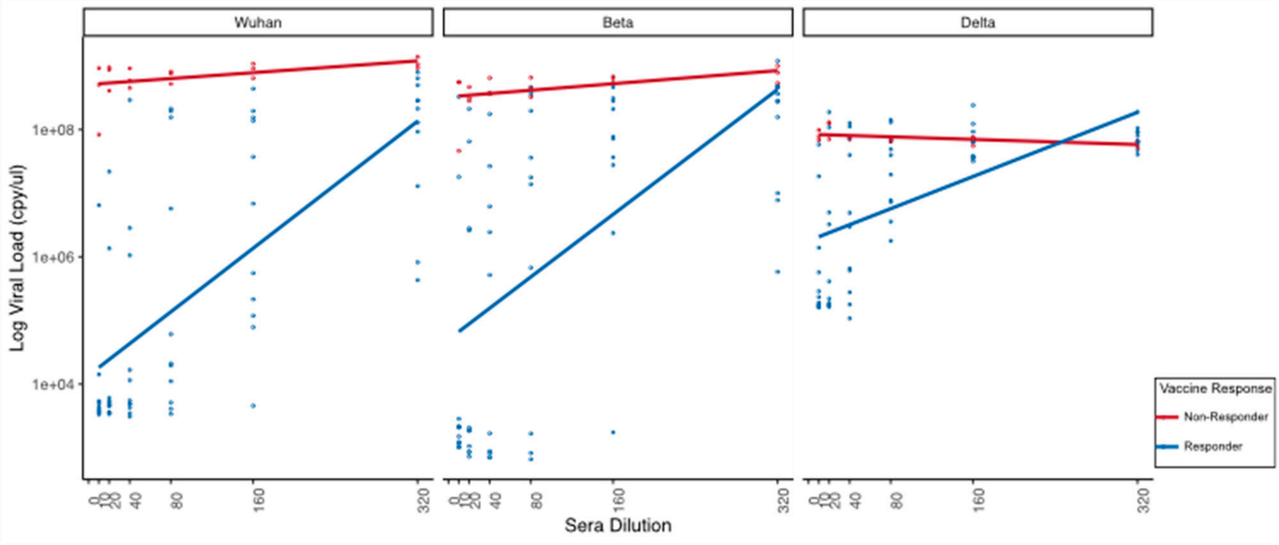

Key:
Beta – (B.1.351) lineage; Delta - (B.1.617.2) lineage;
Non- Responder (n=3) including naïve (n=2) and an immunocompromised patient post 2nd dose of Pfizer-BioNTech (BNT162b2) SARS-CoV-2 vaccination (n=1).
Responders (n= 11) participants who had neutralising antibody titres detected post the 2nd dose of Pfizer-BioNTech (BNT162b2) SARS-CoV-2 vaccination;
Wild type – (A.2.2) lineage;

Supplementary Figure S2: Mutational analysis of fixed mutations and minority allele frequency variants during live virus *in vitro* neutralisation

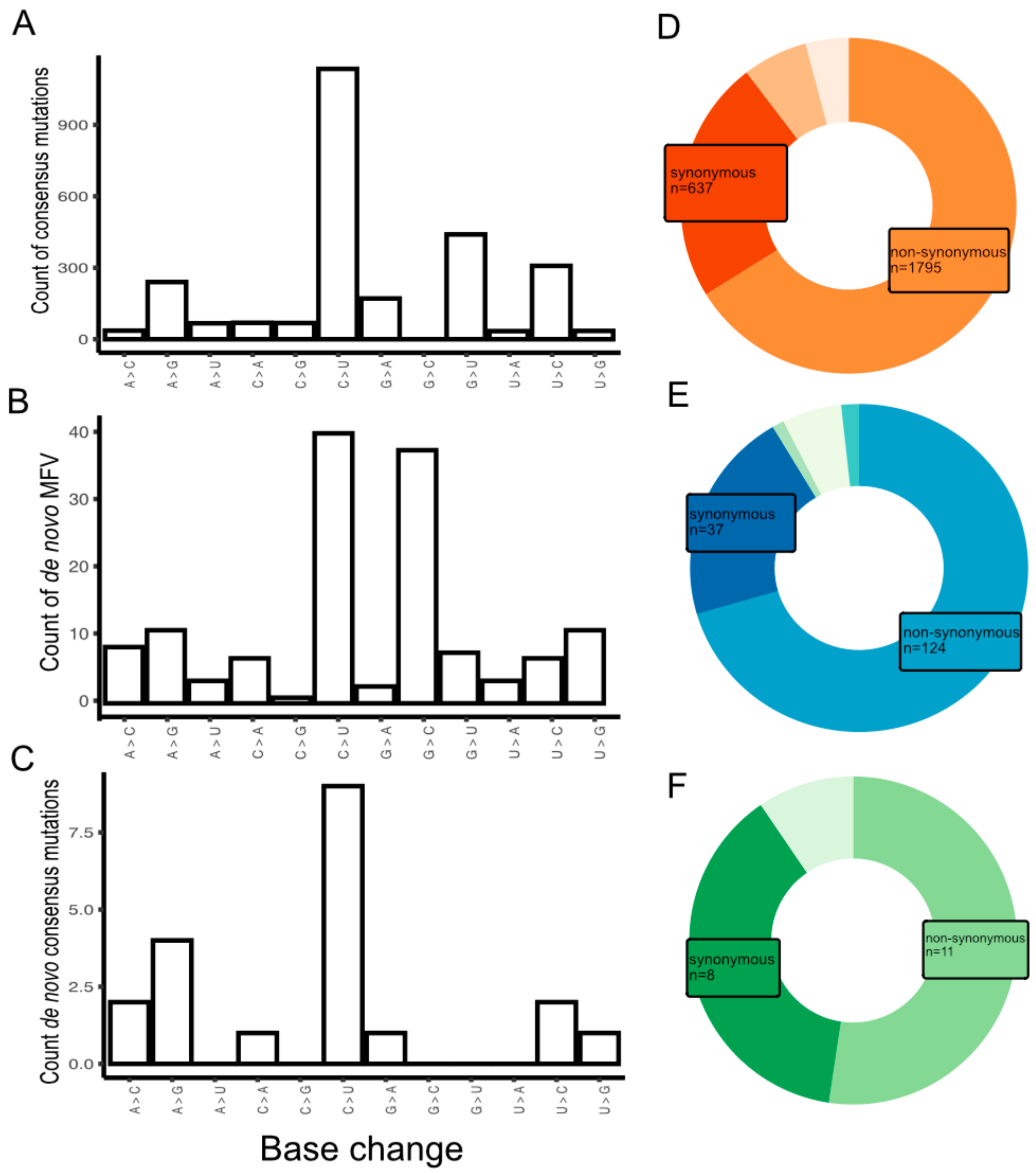

Figure Description: The mutational spectra during live virus neutralisation was quantified. (**A**) All consensus mutations detected against the reference genome. (**B**) *De novo* MFV detected 72 hours post-neutralisation not contained in the inoculating virus. (**C**) The *de novo* consensus mutations detected 72 hours post-neutralisation not detected as a fixed mutation or MFV in the inoculating virus. **(D)** Variant class of consensus mutations detected against the reference genome (indels n=112, noncoding n=170). **(E)** Variant class of *de novo* MFV detected 72 hours post-neutralisation (indels n=10, noncoding n=2, nonsense n=3). **(F)** Variant class of *de novo* consensus mutations detected 72 hours post-neutralisation (noncoding n=2).

Key: MFV – minority allele frequency variant

Supplementary Figure S3: Evolution of minority frequency variants in inoculating viruses 72 hours post-neutralisation.

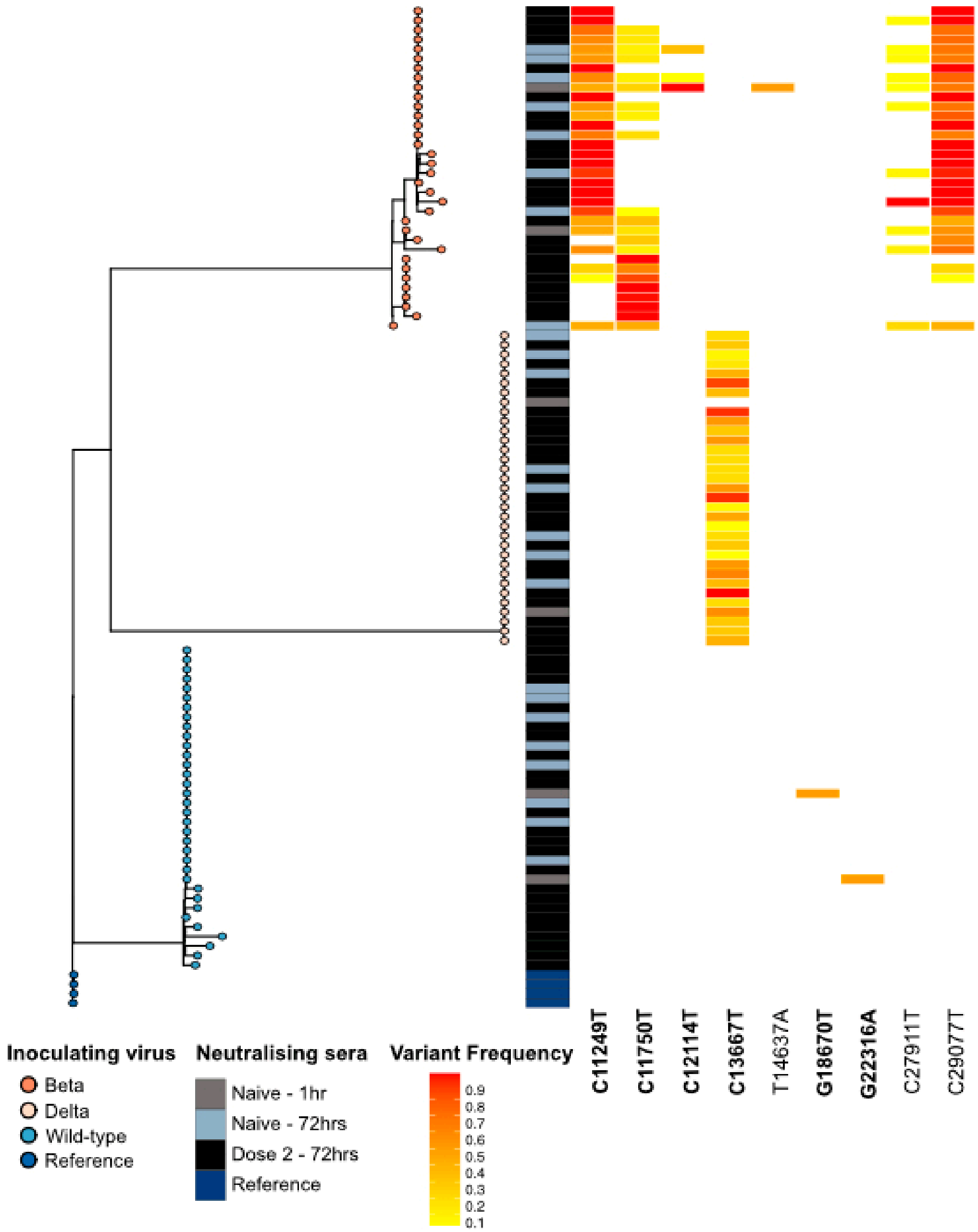

Figure Description: A maximum likelihood phylogeny of the 109 SARS-CoV-2 genomes generated in this study. Tree node colours indicate the lineage of the inoculating virus and the metabar highlights the time of sampling and the vaccine dose of the sera used. *De novo* minority allele frequency variants (MVF) which were detected in the sequence of each inoculating virus at specific genomic locations are shown in the heatmap. The read frequency of these sub-consensus variants is depicted by the colour scale, where a frequency of 0.1 is shown in yellow and a frequency of 0.9 is shown in red.

Key:

Beta – (B.1.351) lineage;

Delta - (B.1.617.2) lineage;

Dose 2 – post the 2nd dose of Pfizer-BioNTech (BNT162b2) SARS-CoV-2 vaccination;

Reference – reference SARS-CoV-2 genome (NCBI GenBank accession MN908947.3);

Wild type – (A.2.2) lineage.
